# Automatic Classification Method of Arrhythmias Based on 12-Lead Electrocardiogram

**DOI:** 10.3390/s23094372

**Published:** 2023-04-28

**Authors:** Xiao Yang, Zhong Ji

**Affiliations:** College of Bioengineering, Chongqing University, Chongqing 400030, China; edencqu@163.com

**Keywords:** 12-lead electrocardiogram, arrhythmias, multimodal features, attention mechanism, automatic classification

## Abstract

Cardiovascular disease is one of the main causes of death worldwide. Arrhythmias are an important group of cardiovascular diseases. The standard 12-lead electrocardiogram signals are an important tool for diagnosing arrhythmias. Although 12-lead electrocardiogram signals provide more comprehensive arrhythmia information than single-lead electrocardiogram signals, it is difficult to effectively fuse information between different leads. In addition, most of the current researches working on automatic diagnosis of cardiac arrhythmias are based on modeling and analysis of single-mode features extracted from one-dimensional electrocardiogram sequences, ignoring the frequency domain features of electrocardiogram signals. Therefore, developing an automatic arrhythmia detection algorithm based on 12-lead electrocardiogram with high accuracy and strong generalization ability is still challenging. In this paper, a multimodal feature fusion model based on the mechanism is developed. This model utilizes a dual channel deep neural network to extract different dimensional features from one-dimensional and two-dimensional electrocardiogram time–frequency maps, and combines attention mechanism to effectively fuse the important features of 12-lead, thereby obtaining richer arrhythmia information and ultimately achieving accurate classification of nine types of arrhythmia signals. This study used electrocardiogram signals from a mixed dataset to train, validate, and evaluate the model, with an average of $F_{1}$ score and average accuracy reached 0.85 and 0.97, respectively. Experimental results show that our algorithm has stable and reliable performance, so it is expected to have good practical application potential.

## 1. Introduction

According to a survey by the World Health Organization, with the changes in lifestyle, cardiovascular diseases have become a major influencing factor endangering human health. Electrocardiogram (ECG) is the most representative and important noninvasive tool for diagnosing cardiac abnormalities. The effectiveness of using a standard 12-lead ECG to diagnose cardiac abnormalities, such as arrhythmia, myocardial infarction, or coronary artery occlusion, has been confirmed in several studies [1]. The traditional ECG analysis method mainly uses experienced cardiovascular disease experts to manually identify the ECG, but this method is manpower-consuming. It has a low prevalence rate, especially in areas with limited medical level, due to many factors such as uneven professional knowledge levels of doctors, different individual differences among patients, and diversity of ECG signals among individuals. As a result, inexperienced doctors are unlikely to judge ECG accurately, which cannot meet people’s medical needs. With the progress of computer hardware technology and algorithms, a large number of scholars at home and abroad have begun to study the automatic classification technology of ECG signals based on Computer Aided Diagnosis Systems (CADS), which can provide doctors with reference and assist them in judging ECG signals [2]. Therefore, in order to bridge the gap between doctors’ professional levels and improve the efficiency and accuracy of medical treatment, it is significant to apply automatic classification technology of ECG signals to clinical diagnosis.

In the past few decades, researchers have proposed a variety of CADS detection technologies. One category is machine learning algorithms based on signal processing techniques and manual extraction of expert features. The general process of such ECG classification algorithms is ECG signal denoising, feature extraction, and training classification models. Many methods in machine learning have been used in the research of ECG classification, such as Support Vector Machine (SVM) [3], Random Forest (RF) [4], K-Nearest Neighbor (KNN) [5], and so on. Hannad et al. used four SVM, two artificial neural networks, and a KNN classifier to categorize ECG signals. They proposed an algorithm based on ECG feature classification and this algorithm extracts 13 types of morphological features from ECG signals. The final average classification accuracy is up to 99%, fully demonstrating its application prospects in clinical environments [6]. Alqudah et al. proposed a new method for detecting and extracting information features from ECG signals based on Gaussian mixture models and wavelet features. Using probabilistic neural networks and random forest classifiers, ECG signals are divided into six categories [7]. Experimental results in the MIT-BIH arrhythmia database show that the combination of Gaussian mixture coefficients and wavelet features provides valuable features related to cardiac performance and can be used as the main feature in arrhythmia classification. Expert features extracted based on clinical knowledge and rule algorithms have good clinical interpretability and can achieve good accuracy in certain ECG category detection. However, these methods are difficult to apply to other categories because some useful key information is lost during ECG segmentation and feature extraction, making it impossible for the model to learn all useful features [8].

Another type is based on end-to-end deep neural networks that do not require manual feature extraction. Researchers have developed various deep learning networks, such as Multilayer Perceptron (MLP), Convolutional Neural Network (CNN), Recursive Neural Network (RNN), Long Short Term Memory (LSTM), and so on. The input of a deep neural network is usually a one-dimensional original ECG signal [9,10,11] and a two-dimensional image [12,13] (a spectral map of the signal or a grayscale image of the drawn signal). The one-dimensional ECG signal is used more frequently. The deep learning model improves the accuracy of different classification tasks, achieving an accuracy similar to that of clinical cardiologists. Currently, CNN is the most popular deep neural network architecture, and has been widely used in computer vision, signal processing, and natural language processing. Its application in cardiology can be traced back more than 20 years [14]. In ECG analysis, it is commonly used for tasks such as the detection of arrhythmia [15] and ST segment changes [16]. Liu W and Huang Q et al. proposed a multi feature branch convolutional neural network (MFB-CNN) based on ECG for automatic detection and localization of ECG signals [17]. He et al. proposed a deep neural network model that combines a deep residual network (ResNet) and a bidirectional long-term and short-term memory network for arrhythmia detection in 12- lead ECG [18]. Yao et al. developed an attention-based time incremental convolutional neural network (ATI-CNN) based on 12-lead ECG data sets, which combines CNN, LSTM networks, and attention mechanisms [19]. Several studies have shown that CNN is an effective classification network for ECG signals [20].

Current researches on automatic diagnostic models for arrhythmia have the following three main shortcomings:

- Due to the limitations of data sets, most classification studies on arrhythmia are conducted on a single data set, and the proposed methods and models cannot be universalized or generalized.
- Most of the researches working on automatic diagnosis of cardiac arrhythmias are based on modeling and analysis of single modal features extracted from one-dimensional ECG sequences, ignoring the frequency domain features of ECG signals.
- Currently, many studies are based on single lead ECG signals, but 12-lead ECG can provide richer arrhythmia information. Different leads can reflect the electrical activity of the heart from different spatial angles, resulting in different leads presenting different shapes at the same time. How to effectively integrate the spatial characteristics of different leads is the focus of current model research.

Therefore, this study has developed a multimodal feature fusion model based on attention mechanism, which implements nine types of arrhythmia classification on a mixed dataset, including eight types of abnormal ECG and normal sinus rhythm. The main research content of this article is as follows:

- In this paper, multimodal features of ECG signals are extracted for classification of arrhythmia signals. A dual channel network is used to extract the one-dimensional time series features and two-dimensional time–frequency features of the 12-lead ECG signals, and then the feature fusion layer is used to integrate the two modal features.
- In this paper, a CNN is used as a feature extraction network. In order to efficiently learn the features in one-dimensional ECG sequences and two-dimensional ECG time–frequency maps, stacked one-dimensional and two-dimensional bottleneck residual structures are used as feature extraction networks. The bottleneck residual structure has the advantages of increasing network depth, enhancing feature transfer, and reducing the number of parameters. Therefore, using the bottleneck residual structure can improve the learning ability of deep neural networks.
- In this paper, attention mechanism is combined with dual channel deep neural networks to preserve the useful information in one-dimensional ECG sequences and two-dimensional ECG time–frequency maps as fully as possible, reducing the loss of valuable information. For one-dimensional ECG sequences, introducing channel domain attention mechanisms can make one-dimensional ECG sequences pay more attention to the relationships between different channels. By aggregating the characteristic relationships between channels, the characteristic values between each channel can be adaptively adjusted. A mixed domain attention mechanism is introduced for two-dimensional time–frequency electrocardiograms, which enables the two-dimensional bottleneck residual structure to simultaneously enhance important features in both channels and spatial domains. That is, it can not only strengthen important channel features and suppress unwanted channel features, but also efficiently learn areas that require special attention in two-dimensional time–frequency electrocardiograms to preserve key features in the time–frequency diagram. Experiments have shown that introducing attention mechanisms can better enable feature extraction networks to focus on key feature locations in the ECG band.

## 2. Materials and Methods

### 2.1. Dataset

**Figure 1:**
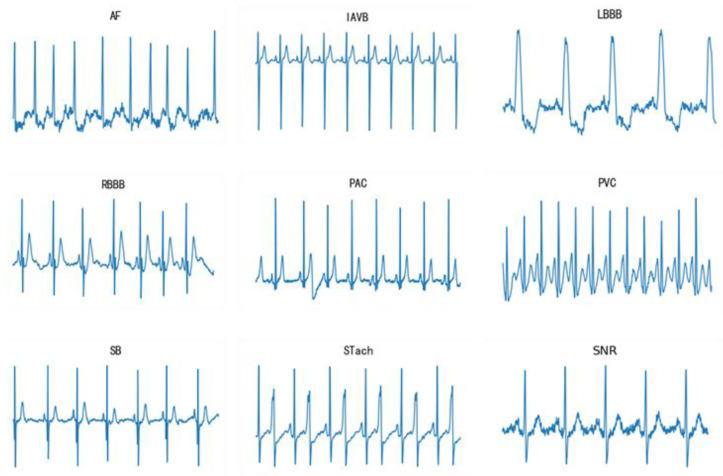
Samples of each class of ECG.

This paper focuses on classifying 9 classes of ECG signals, which are Atrial fibrillation (AF), First-degree atrioventricular block (IAVB), Left bundle branch block (LBBB), Right bundle branch block (RBBB), Premature atrial contraction (PAC), Premature ventricular contractions (PVC), Sinus Bradycardia (SB), Sinus Tachycardia (Stach), Normal (SNR). Figure 1 shows the samples from the 9 ECG signal classes.

Various researches on disease classification based on ECG signals require the support of ECG data sets, and the quality and quantity of data restrict the final classification results. There are a large number of publicly available datasets for researchers to use. Currently, most cardiovascular disease classification models are tested on a single dataset, and the models perform well on a single dataset, while they perform poorly on other datasets. The experimental results indicate that the application of the models has limitations and the models are not robust. Therefore, this article combines data from different sources for training, through continuous correction of a large amount of data, and finally obtains a robust model. The used dataset in this work combines three public databases containing 13,431 recordings of 12-lead ECG. This type of ECG is the most used in clinical cases because of the large amount of information that it generates. These recordings are sampled at a frequency of 500 Hz. Table 1 describes the characteristics of each database. The used dataset in this work contains 9 classes, where 8 classes are of CVDs and a class represents a normal heart state. Table 2 shows the distribution of these classes on each database.

### 2.2. Proposed Method

#### 2.2.1. Two-Dimensional Electrocardiogram Time–Frequency Diagram

**Figure 2:**
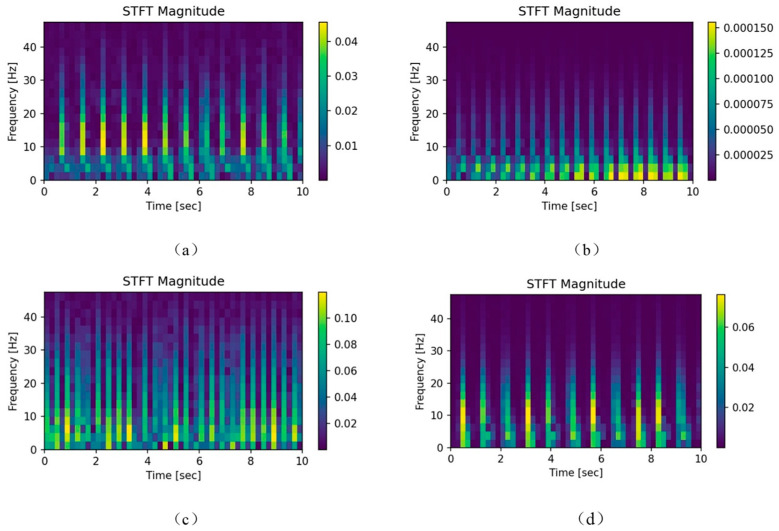
Time-spectrum images corresponding to four different arrhythmia of I-lead ECG. (**a**) SNR; (**b**) RBBB; (**c**) AF; (**d**) IAVB.

Short-time Fourier Transform(STFT) is a commonly used tool in signal processing. It is derived from discrete Fourier transform and can represent both time and frequency domain characteristics of signals. Select different types of ECG records and use STFT to convert the timing signals of I-lead ECG, the visualization results of time–frequency spectrum images corresponding to I-lead ECG signals of SNR, RBBB, AF, and IAVB are shown in Figure 2. It can be seen that there are significant differences between time–frequency spectrum images of different arrhythmia, With the different time–frequency characteristic information, convolutional neural networks can be used to extract features and classify them, thereby achieving the goal of automatic diagnosis of arrhythmia.

There are two key parameters to be determined when using STFT, namely, the window function and the window function length. In time–frequency analysis, Hanning window is generally selected as the window function. The main lobe of this window is widened and reduced. Although the frequency resolution decreases, the side lobe is significantly reduced, which can effectively suppress spectrum leakage. Therefore, Hanning window is selected to analyze the time–frequency diagram of ECG signals. After selecting the analysis window function, it is necessary to determine the length of the window function. When the window length is 256, good signal time–frequency resolution performance can be obtained in both the frequency domain and the time domain.

#### 2.2.2. Channel Domain Attention Module

**Figure 3:**
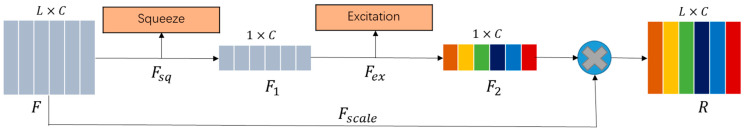
CA module structure diagram.

The channel domain attention (CA) mechanism focuses on the impact of the feature vectors on each channel on the classification results. Through CA module, you can learn the corresponding weight of each channel. The greater the weight, the more important the channel information is. The representative method is Squeeze and Extension Networks (SENet), which was proposed by Jie Hu et al. [22]. SENet is not a complete network structure, but a sub-structure that can be easily embedded into other classification or detection models. In this study, SENet is used to enhance important features in one-dimensional ECG sequences, so it is necessary to make corresponding improvements to SENet to obtain a one-dimensional channel domain attention module. The structure diagram of the channel domain attention module used in this article is shown in Figure 3.

#### 2.2.3. Hybrid Domain Attention Mechanism

**Figure 4:**
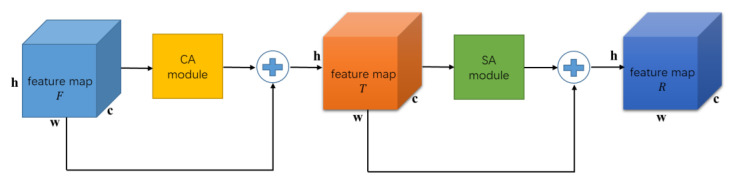
HA module structure diagram.

The hybrid domain attention (HA) module connects the spatial domain attention (SA) and CA modules in a serial manner. Taking a 12-lead two-dimensional ECG time–frequency map as an example, not all regions on the time–frequency map contain useful feature information. The SA mechanism utilizes convolutional neural networks to efficiently learn regions that require special attention, transform the spatial information from the original time–frequency map into another space to preserve key features in the time–frequency map. The HA module compensates for the lack of attention in both the spatial and channel domains of the two-dimensional time–frequency map by simultaneously adding attention to both the spatial and channel domains, enabling convolutional neural networks to simultaneously enhance important features in both the spatial and channel domains. The structure of the hybrid attention module HA designed in this article is shown in Figure 4.

**Figure 5:**
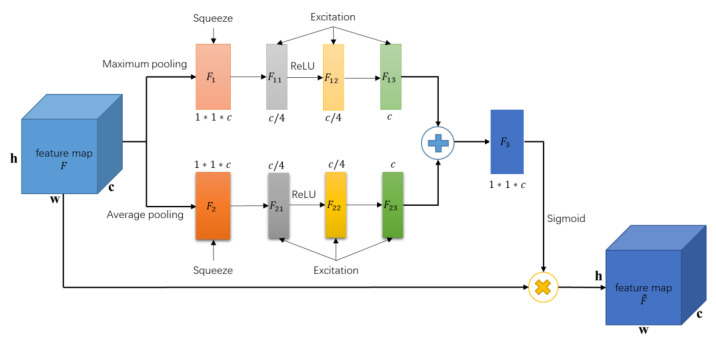
CA module structure diagram in the HA mechanism.

The structure diagram of the CA module in the HA mechanism (H-CA) is shown in Figure 5, including two independent pooling operation branches: global maximum pooling and global average pooling. The input feature map F is squeezed and excited in parallel to obtain the attention weight map $F_{13}$ and $F_{23}$, respectively. To obtain the two-pooling information, $F_{13}$ and $F_{23}$ are fused by summing the features to obtain the attention weight map $F_{3}$. Finally, $F_{3}$ is point multiplied with the input feature map F to obtain the output feature map $\tilde{F}$ enhanced by attention in the channel domain.

**Figure 6:**
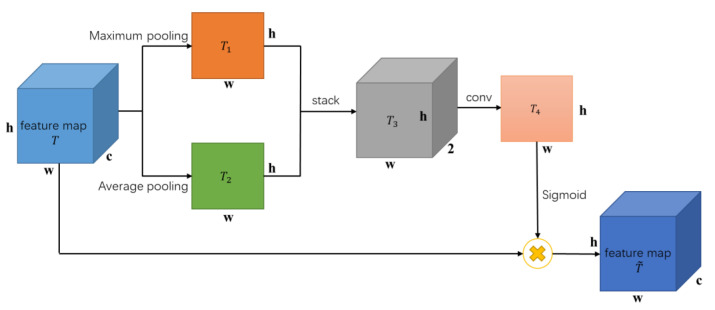
SA module structure diagram in the HA mechanism.

The structure diagram of the SA module in the HA mechanism (H-SA) is shown in Figure 6. The H-SA module also includes a maximum pooling branch and an average pooling branch. Both pooling operations generate feature maps $T_{1}$ and $T_{2}$ of the same size, and then stack $T_{1}$ and $T_{2}$ to obtain feature map $T_{3}$. $T_{3}$ is first convolved and then normalized using the Sigmoid function to obtain the attention weight map $T_{4}$. $T_{4}$ is point multiplied with the input feature map *T* to obtain the output feature map $\tilde{T}$, which is the feature map after learning to pay attention to the spatial domain.

#### 2.2.4. Model Structure

ECG records the electrical activity of human heart, which can reflect many important disease information and has diagnostic value for arrhythmias. One-dimensional ECG sequences mainly reflect the characteristics of ECG signals from the time domain perspective, and most of the current research works on automatic diagnosis of arrhythmias are analyzed by single-mode features extracted from one-dimensional ECG sequences. Based on the non-stationarity and complexity of ECG signals, the extraction of multimodal features is beneficial to optimize the analysis results. Time–frequency analysis is considered as the preferred method for analyzing non-stationary signals, which extracts the potential features of the signal through a two-dimensional time–frequency function. The two-dimensional ECG time–frequency map is obtained based on the STFT, which reflects the time–frequency domain characteristics of the ECG signal. Therefore, a dual-channel multimodal feature fusion network is used to extract features of different dimensions from 1D ECG sequences and 2D ECG time–frequency maps, respectively, and then feature fusion is performed.

**Figure 7:**
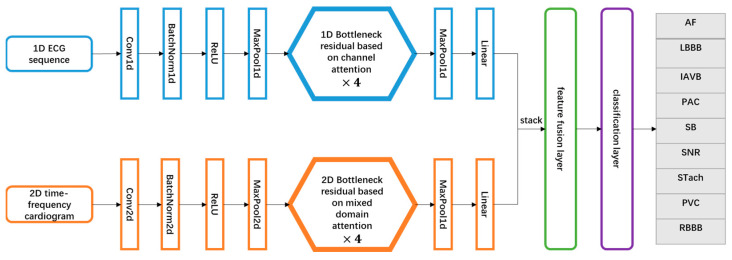
The proposed network architecture.

The attention mechanism is a technique to enhance the extraction of important features. It generates different weights through model training, and then assigns larger weights to features with greater discriminative power and, conversely, smaller weights to irrelevant or distracting features. It helps deep neural networks to identify critical information more accurately. Therefore, in order to accurately classify arrhythmia signals, the attention mechanism is combined with a dual-channel multimodal feature fusion network to construct an end-to-end trainable deep neural network, namely Electrocardiogram Classification Network based on Multi-model information and Attention Mechanism (EC-MAM). The model architecture is shown in Figure 7.

**Figure 8:**
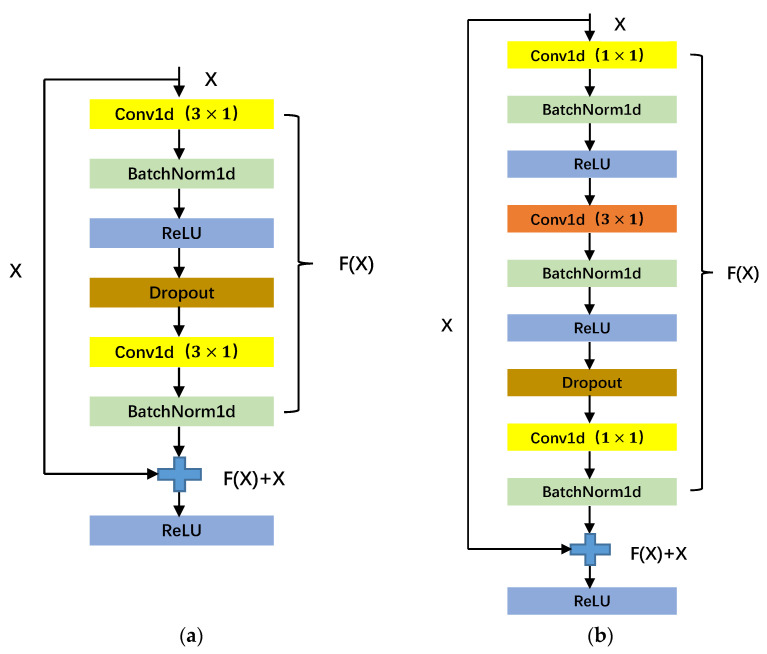
(**a**) Residual structure; (**b**) Bottleneck residual structure.

In this paper, 1D CNN and 2D CNN are used to extract one-dimensional ECG sequence features and two-dimensional ECG time–frequency map features, respectively. In order to improve the learning ability of deep neural networks, stacked convolutional pooling layers are used to extract the characteristics of ECG signals in the proposed EC-MAM model. However, due to the existence of nonlinear activation functions, each input to output process is almost irreversible, which also leads to many irreversible information losses. The deepening of the network is prone to the problem of gradient disappearance. The idea of residual networks is to solve the problem that the performance of deep neural networks becomes saturated or even degraded with the increase of depth. As shown in Figure 8a, the input of the residual block is X, the output is H(X) = F(X) + X, and the residual term is F(X). Learning F(X) = 0 is easier than learning H(X) = X because the parameters in each layer of the network are generally initialized with a bias toward 0 and the ReLU activation function is able to activate negative numbers to 0. This shows that the residual block is able to deepen the network in such a way that the performance of the deep network is at least equal to that of the shallow network, giving the network layer the ability to have constant mapping. The bottleneck residual structure is a special residual structure, as shown in Figure 8b. The core idea of the bottleneck residual structure is to use multiple 1 × 1 convolutional kernels instead of large convolutional kernels, so compared with the ordinary residual structure, the bottleneck residual structure uses 1 × 1 convolutional kernels to make the original number of channels decrease and then increase, and the number of channels decrease can be more effective and intuitive for data training and feature extraction. Therefore, the bottleneck residual structure has the advantages of increasing network depth, enhancing feature transfer and reducing the number of parameters.

**Figure 9:**
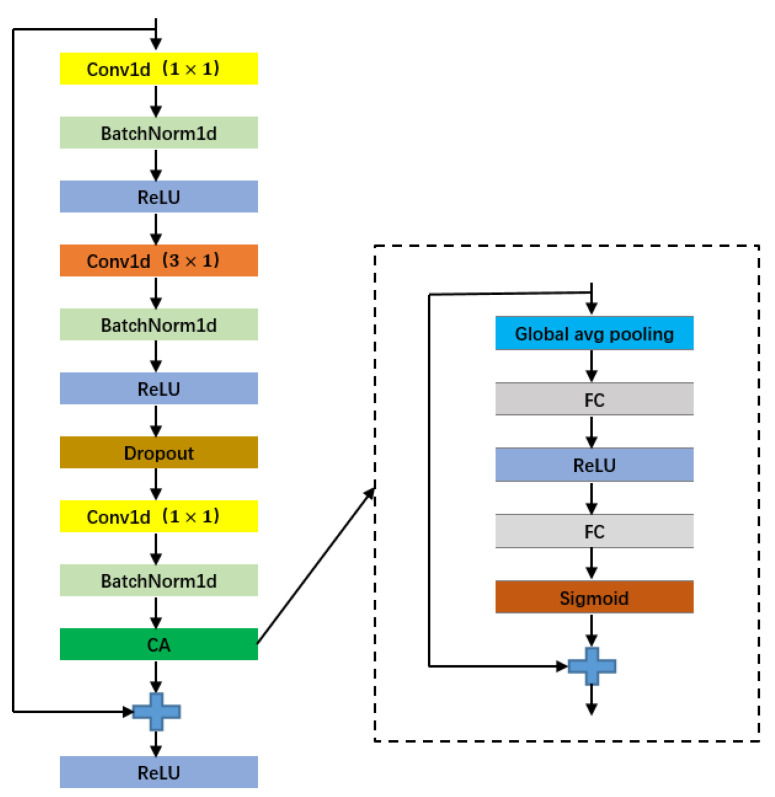
1D bottleneck residual structure based on channel domain attention mechanism.

**Figure 10:**
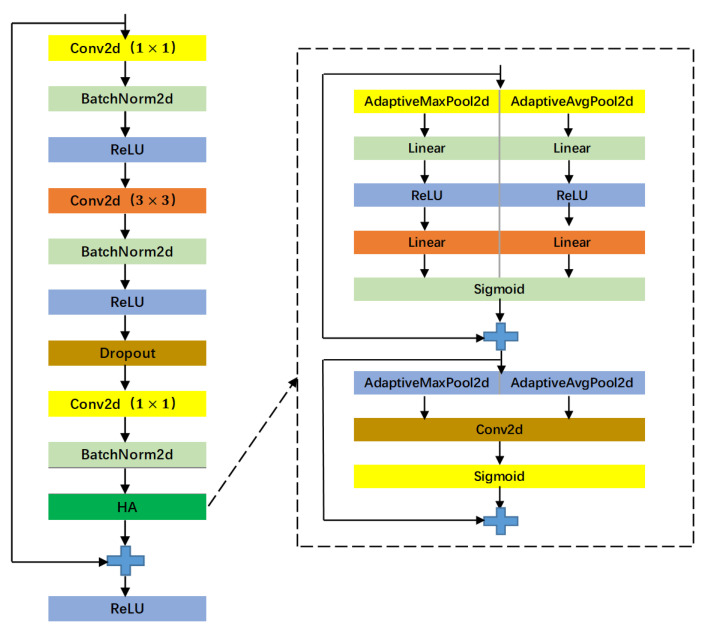
2D bottleneck residual structure based on mixed domain attention mechanism.

However, the convolution operation in the bottleneck residual structure is to fuse features of a local region. By improving the receptive field to fuse more features, the feature map generated through the convolution operation cannot utilize relevant information outside this region. In order to obtain global information, this article introduces an attention module based on the bottleneck residual structure, as shown in Figure 9. This figure is a one-dimensional bottleneck residual structure with a CA module added to extract the characteristics of one-dimensional ECG sequences; Figure 10 shows a two-dimensional bottleneck residual structure with a HA module added to extract two-dimensional time–frequency ECG features.

## 3. Results

### 3.1. Model Input

The input of EC-MAM is a parallel dual input, which includes two parts: a one-dimensional ECG time series and a two-dimensional ECG time–frequency map. In order to reduce the interference of noise on model training, an improved wavelet threshold algorithm is used to remove the baseline drift and EMG interference with a wide frequency distribution range, and a notch filter is used to remove fixed frequency power frequency interference.

For one-dimensional ECG time series, this part conducts the following processing based on the characteristics of signal samples in mixed data sets: In mixed data sets, the length of ECG samples varies from 6 s to 60 s, with a median of 30 s. Since the CNN model receives signals with a constant length, signals shorter than 30 s are zeroed in, and signals longer than 30 s are truncated to the last 30 s. The sampling frequency of the ECG signal is 500 Hz, therefore, the input format of one-dimensional ECG time series is $X_{1}\in R^{12\times 15,000}$.

For two-dimensional ECG time–frequency maps, in order to comprehensively obtain the hidden pathological feature information in each lead, all conversion of 12-lead signals have been conducted, and the resolution of each time–frequency map is uniformly set to 224 × 224. Therefore, the input of two-dimensional ECG time–frequency map is $X_{2}\in R^{12\times 224\times 224}$.

The disease classification on the mixed dataset is unbalanced, which will lead to the poor effect of the model on disease classification with a small number of samples. Therefore, the weighted multi-label cross entropy calculated based on the category frequency is used as the loss function, and its calculation formula is as follows:

(1) $$L=\sum_{i=1}^{9}-w_{i}[y_{i}\log\left(\hat{y_{i}}\right)+(1-y_{i})\log\left(1-\hat{y_{i}}\right)],$$

where i represents one of the nine categories of arrhythmia diseases, $y_{i}$ is the true label for the disease classification of the sample, $\hat{y_{i}}$ is the corresponding model prediction probability, and $w_{i}$ is the weight of the classification category, $w_{i}$ is calculated from the positive sample frequency of each classification in the training set, and its calculation formula is as follows:

(2) $$w_{i}=\frac{1}{\log{(n}_{i})}$$

where $n_{i}$ represents the number of samples on the training set where disease classification is a positive case. It can be seen that the more positive samples in disease classification, the corresponding classification weight will be relatively reduced, while the weight of diseases with fewer positive samples will be relatively increased. By increasing the punishment for disease classification errors with fewer positive examples, it helps the model pay attention to the problem of sample imbalance in the dataset, thereby improving the average performance of the model.

As mentioned in Section 2.1 the dataset used comprises 13,431 ECG records. First, dataset was split into two sets, test set and training and validation set, in the ratio of 0.5:0.5. After this, five-fold cross-validation approach on the training and validation set was applied. The dataset was randomly divided into five folds. At each round, four folds out of five folds were used for training, one-fold for validation.

### 3.2. Hyperparameters

In this experiment, the parameters of the convolutional layer and the fully connected layer are randomly initialized. The training model is optimized using the Adam optimizer, applying the cross-entropy loss function as the loss metric function of the model, initializing the learning rate at 0.001, and setting the dropout parameter in the model to 0.4. An automatic learning rate adjustment strategy is adopted to prevent model overfitting during model training. After ten iterations, the learning rate decreases to 50% of the original. The experiment trained a total of 50 cycles, with the batch size set to 64.

### 3.3. Evaluation Metrics

The evaluation indicators used in this article include Precision, Recall, $F_{1}$ score, ROC, and Accuracy. These indicators are used to evaluate the effectiveness of the model. The higher the score, the better the classification effect of the model. The definitions of these indicators are as follows:

(3) $${Precision}_{x}=\frac{{TP}_{x}}{{TP}_{x}+{FP}_{x}}$$

(4) $${Recall}_{x}=\frac{{TP}_{x}}{{TP}_{x}+{FN}_{x}}$$

(5) $${Accuracy}_{x}=\frac{{TP}_{x}+{TN}_{x}}{{TP}_{x}+{TN}_{x}+{FP}_{x}+{FN}_{x}}$$

(6) $$F_{1x}=\frac{2\times ({Precision}_{x}\times {Recall}_{x})}{{Precision}_{x}+{Recall}_{x}}$$

where, *x* = 1, 2, …, 9, ${TP}_{x}$ represents the number of samples for the correctly predicted category, ${TN}_{x}$ indicates the number of samples of non category that are correctly predicted, ${FN}_{x}$ is the number of samples in the category of prediction error, ${FP}_{x}$ represents the number of samples of non-category *x* with prediction errors. To better evaluate the performance of multi-label classification, we adopt mean score of each metric on nine classes (1 normal and 8 abnormal). Mean $F_{1}$ score is used to select the best-performing model.

### 3.4. Experimental Results

As shown in Table 3, the proposed model has achieved good performance in the multi-label arrhythmia classification task. For nine types of arrhythmia, the mean of $F_{1}$ score reached 85%, the mean of accuracy rate reached 96.7%, and the mean of accuracy rate and recall rate were both above 84%. When identifying arrhythmia signals in three categories of AF, SB, and SNR, $F_{1}$ scores were above 0.9.

**Figure 11:**
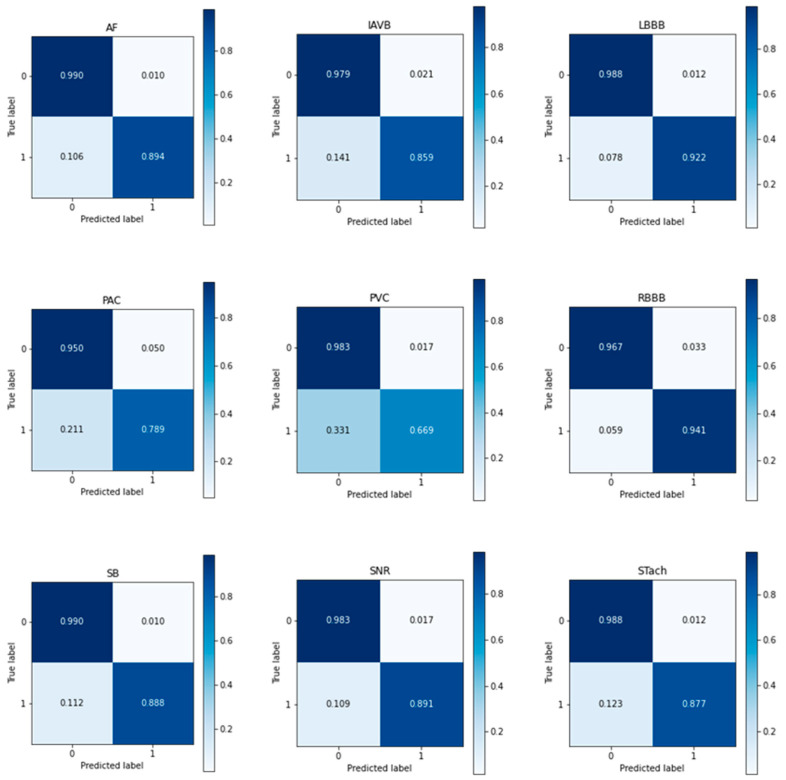
Multi-label confusion matrices.

In order to demonstrate the effectiveness of the proposed EC-MAM model on different arrhythmia signal recognition tasks more intuitively, a confusion matrix with nine classifications for arrhythmia signals was calculated on the test set, as shown in Figure 11. From Figure 11, it can be observed that the degree of difficulty in classifying different arrhythmia signals is PVC, PAC, IAVB, STach, SB, SNR, AF, LBBB, and RBBB, in descending order. The diagnosis of AF, LBBB, and RBBB has a low false positive rate and false negative rate. For example, the false negative rate of classified RBBB is 0.059, and the false positive rate is 0.033, indicating that the EC-MAM model has a good classification effect for these three types of diseases, and has a low misdiagnosis rate. However, from the confusion matrix, it is shown that EC-MAM model performs poorly in classifying PVC, PAC, and IAVB. Overall, the false negative rate and the false positive rate for classifying these three types of arrhythmia signals are higher. For example, the false negative rate for classifying PVC is as high as 0.331, and the false positive rate is as low as 0.017. This indicates that the sensitivity of the EC-MAM model in classifying PVC, PAC, and IAVB is relatively low. If the sensitivity of a diagnostic experiment is relatively low, there will be many false negative patients. This can delay patient visits, which will subsequently affect the progression and recovery of the disease, and even lead to premature death.

Params refer to the total number of parameters that need to be trained in model training, independent of the input data and mainly related to the structure of the model. They are used to calculate the spatial complexity of the model, since the number of parameters in many models is too large, it is generally measured in megabytes (M). The model storage space not only includes parameter quantities, but also includes network architecture information and optimizer information, generally measured in Mbyte (MB). Floating point Operations (FLOPS) represent the number of floating-point operations per second and are a measure of hardware performance. Generally, Giga Floating point Operations Per Second (GFLOPS) is used to represent it, and its unit is usually G (1 GFLOPS = 1000 MFLOPS). The resource consumption of the EC-MAM model is shown in Table 4.

### 3.5. Ablation Experiments

In order to verify the effectiveness of the proposed EC-MAM model, two sets of ablation experiments were designed. The first set of ablation experiments was used to verify the effectiveness of multimodal features in the EC-MAM model in improving model performance; The second set of ablation experiments was used to verify the effectiveness of the attention mechanism in improving the performance of the model. For different models, ensure consistent data set partitioning, that is, based on the same training set, verification set, and test set.

1. The first group of ablation experiments

The first model is Electrocardiogram Classification Network based on Multi-model information and Attention Mechanism (EC-MAM): a multimodal ECG classification network based on attention mechanism. The model combines one-dimensional time series characteristics and two-dimensional time–frequency characteristics of 12-lead ECG signals.

The second model is One Dimensional Electrocardiogram Classification Network based on Single mode information and Attention Mechanism (1D-EC-SAM): a single mode one-dimensional ECG classification network based on attention mechanism. This model only extracts one-dimensional ECG time series features.

The third model is Two-Dimensional Electrocardiogram Classification Network based on Single mode information and Attention Mechanism (2D-EC-SAM): a single mode two-dimensional ECG classification network based on attention mechanism. This model only extracts two-dimensional time–frequency characteristics of ECG.

In the first set of ablation experiments, the above three models were constructed. As shown in Table 5, the performance of 12-lead arrhythmia classification based on different modal characteristics is compared. By comparing the mean *F*_1_ scores of the three models in nine disease classifications. it can be seen that EC-MAM > 1D-EC-SAM > 2D-EC-SAM, the single-mode model that only extracts one-dimensional ECG time series features has better classification performance than the single-mode model that only extracts two-dimensional ECG time–frequency map features on most disease types. However, the proposed multimodal feature model EC-MAM has the best classification performance in multi-label arrhythmia classification tasks, which verifies the effectiveness of fusing multimodal features to improve model performance.

2. The second group of ablation experiments

The fourth model is Electrocardiogram Classification Network based on multi-mode information (EC-MMI): Multimodal ECG Classification Network. Compared with the first model, this model eliminates the attention mechanism in dual channel networks. The purpose of this model is to verify the effectiveness of attention mechanisms for improving model performance.

The comparison results are shown in Table 6. The classification performance of the model with adding attention mechanism has been greatly improved compared to the model without adding attention. In the classification of nine diseases, the mean $F_{1}$ score increased by two percentage points. According to the analysis in Table 6, the attention mechanism mainly improves the recognition accuracy of PAC and PVC, and many models have difficulties in classifying PAC and PVC. On the one hand, there may be a small number of PAC and PVC samples in the mixed dataset, resulting in data imbalance, making it difficult for the model to classify the two types of signals; On the other hand, the pathological characteristics of PAC and PVC on the ECG are similar to other types of arrhythmia diseases, which also lead to errors in the classification of PAC and PVC in the model. Therefore, deep neural networks with increased attention mechanisms have stronger feature extraction capabilities, which can automatically identify more discriminative hidden features from data, and make more accurate predictions of easily confused arrhythmia signals.

However, EC-MAM uses a two-channel network to fuse multimodal features, and this approach may cause an increase in model complexity, with the consequent increase in the ability of the model to fit the training data, resulting in overfitting. Due to the varying characteristics of signals, the classification ability of EC-MAM for multi-label signal classification will vary for different signals. The multimodal model EC-MAM does not perform as well as the unimodal models 1D-EC-SAM, 2D-EC-SAM, and EC-MMI when classifying the three signals LBBB, SNR and Stach.

## 4. Conclusions

This paper mainly studies the automatic arrhythmia detection task based on 12-lead ECG, and develops a multimodal feature fusion model based on attention mechanism. Compared with the traditional ECG classification model, there are three main improvements. One improvement is to use a dual channel network to extract one-dimensional time series features and two-dimensional time–frequency features of 12-lead ECG signals, and then use a feature fusion layer to integrate the two modal features. the second improvement is to add different attention mechanisms to the dual channel network to extract more discriminative features from different perspectives, that is, by giving different channels or regions different weights, flexibly capturing global and local relationships, allocating more attention to key feature areas, and allocating less attention to noise areas, thereby achieving enhancement of key features and noise reduction. The attention mechanism effectively reduces the interference of ECG signal noise on arrhythmia recognition, enabling the model to extract more robust features, and improving model classification performance. The last improvement is that the article combines data from different sources for training, and through continuous correction of a large amount of data, finally obtains a model with strong robustness. The experimental results indicate that the average values of reducing false results of the proposed automatic algorithm is 3.3% and the model has good classification performance, which proves that the model has universality and strong generalization ability.

Because the classification model proposed in this paper is a dual-channel network, the model has a large number of parameters, a large volume, and a large computational time and spatial complexity. Therefore, in the future, lightweight networks with smaller size and less computational complexity will be studied to achieve higher classification accuracy with less resource consumption. Through further improvement and research, the performance of the model can be further improved, and the algorithm can be applied to practical tasks earlier.

## Figures and Tables

**Table 1:** Description of each database’s characteristics.

Database	Sources	Number of ECG Recordings	Length of Every ECG Recording
CPSC2018 [21]	China Physiological Signal Challenge in 2018	5806	6–60 s
CPSC2018-Extra [21]		920	6–60 s
Georgia [21]	Georgia	6705	10 s

**Table 2:** Pathologies distribution in each database.

Type of Arrhythmia	CPSC2018	CPSC2018-Extra	Georgia	Total
AF	1176	148	570	1894
IAVB	716	105	769	1590
LBBB	228	37	231	496
RBBB	1828	112	556	2496
PAC	609	121	640	1370
PVC	698	187	395	1280
SB	0	45	1677	1722
Stach	0	299	1261	1560
SNR	907	4	1752	2663

**Table 3:** Twelve-lead model performance averaged on 5-fold tests.

Classes	Precision	Recall	$F_{1}$	AUC	ACC
AF	0.928	0.891	0.909	0.980	0.977
LBBB	0.883	0.800	0.831	0.976	0.988
IAVB	0.854	0.879	0.865	0.980	0.966
PAC	0.673	0.737	0.702	0.926	0.936
SB	0.933	0.913	0.923	0.992	0.980
SNR	0.895	0.925	0.909	0.987	0.963
STach	0.876	0.907	0.891	0.987	0.974
PVC	0.845	0.638	0.726	0.938	0.953
RBBB	0.892	0.905	0.898	0.983	0.963
Mean	0.864	0.844	0.850	0.972	0.967

**Table 4:** Model resource consumption data.

Model	Params (M)	GFLOPS (G)	Storage (MB)
EC-MAM	57.19	80.27	218

**Table 5:** Performance of the first ablation experimental model.

Classes	EC-MAM	1D-EC-SAM	2D-EC-SAM
AF	0.909	0.898	0.805
LBBB	0.831	0.848	0.851
IAVB	0.865	0.836	0.731
PAC	0.702	0.681	0.618
SB	0.923	0.921	0.918
SNR	0.909	0.909	0.868
STach	0.891	0.911	0.892
PVC	0.726	0.700	0.696
RBBB	0.898	0.885	0.832
Mean	0.850	0.843	0.801

**Table 6:** Performance of the second ablation experimental model.

Classes	EC-MMI	EC-MAM
AF	0.890	0.909
LBBB	0.851	0.831
IAVB	0.855	0.865
PAC	0.636	0.702
SB	0.913	0.923
SNR	0.919	0.909
STach	0.908	0.891
PVC	0.683	0.726
RBBB	0.866	0.898
Mean	0.836	0.850

## Data Availability

The data presented in this study are openly available at Alday, E.A.P. et al. [22].
